# Constitutional mismatch repair-deficiency: current problems and emerging therapeutic strategies

**DOI:** 10.18632/oncotarget.26249

**Published:** 2018-10-23

**Authors:** Malak Abedalthagafi

**Affiliations:** ^1^ Genomics Research Department, Saudi Human Genome Project, King Fahad Medical City, King Abdulaziz City for Science and Technology, Riyadh, Saudi Arabia; ^2^ Department of Pathology, Brigham and Women's Hospital, Harvard Medical School, Boston, MA, USA

**Keywords:** CMMRD, childhood cancer, mismatch repair, immunotherapy, predisposition syndrome

## Abstract

Mismatch repair (MMR) proteins remove errors from newly synthesized DNA, improving the fidelity of DNA replication. A loss of MMR causes a mutated phenotype leading to a predisposition to cancer.

In the last 20 years, an increasing number of patients have been described with biallelic MMR gene mutations in which MMR defects are inherited from both parents. This leads to a syndrome with recessive inheritance, referred to as constitutional mismatch repair-deficiency (CMMRD). CMMRD is a rare childhood cancer predisposition syndrome. The spectrum of CMMRD tumours is broad and CMMRD-patients possess a high risk of multiple cancers including hematological, brain and intestinal tumors. The severity of CMMRD is highlighted by the fact that patients do not survive until later life, emphasising the requirement for new therapeutic interventions.

Many tumors in CMMRD-patients are hypermutated leading to the production of truncated protein products termed neoantigens. Neoantigens are recognized as foreign by the immune system and induce antitumor immune responses. There is growing evidence to support the clinical efficacy of neoantigen based vaccines and immune checkpoint inhibitors (collectively referred to as immunotherapy) for the treatment of CMMRD cancers. In this review, we discuss the current knowledge of CMMRD, the advances in its diagnosis, and the emerging therapeutic strategies for CMMRD-cancers.

## INTRODUCTION

DNA mismatch repair (MMR) deficiency is a well characterised form of genetic instability in cancer [1–5], characterized by a failure to repair DNA replication-associated errors. A defective MMR system leads to the persistence of mismatched mutations across the genome, particularly in regions of repetitive DNA (microsatellites), leading to microsatellite instability (MSI). MSI causes the production of truncated protein products, resulting in the development of life-threatening malignancies [1–9].

**Figure 1:**
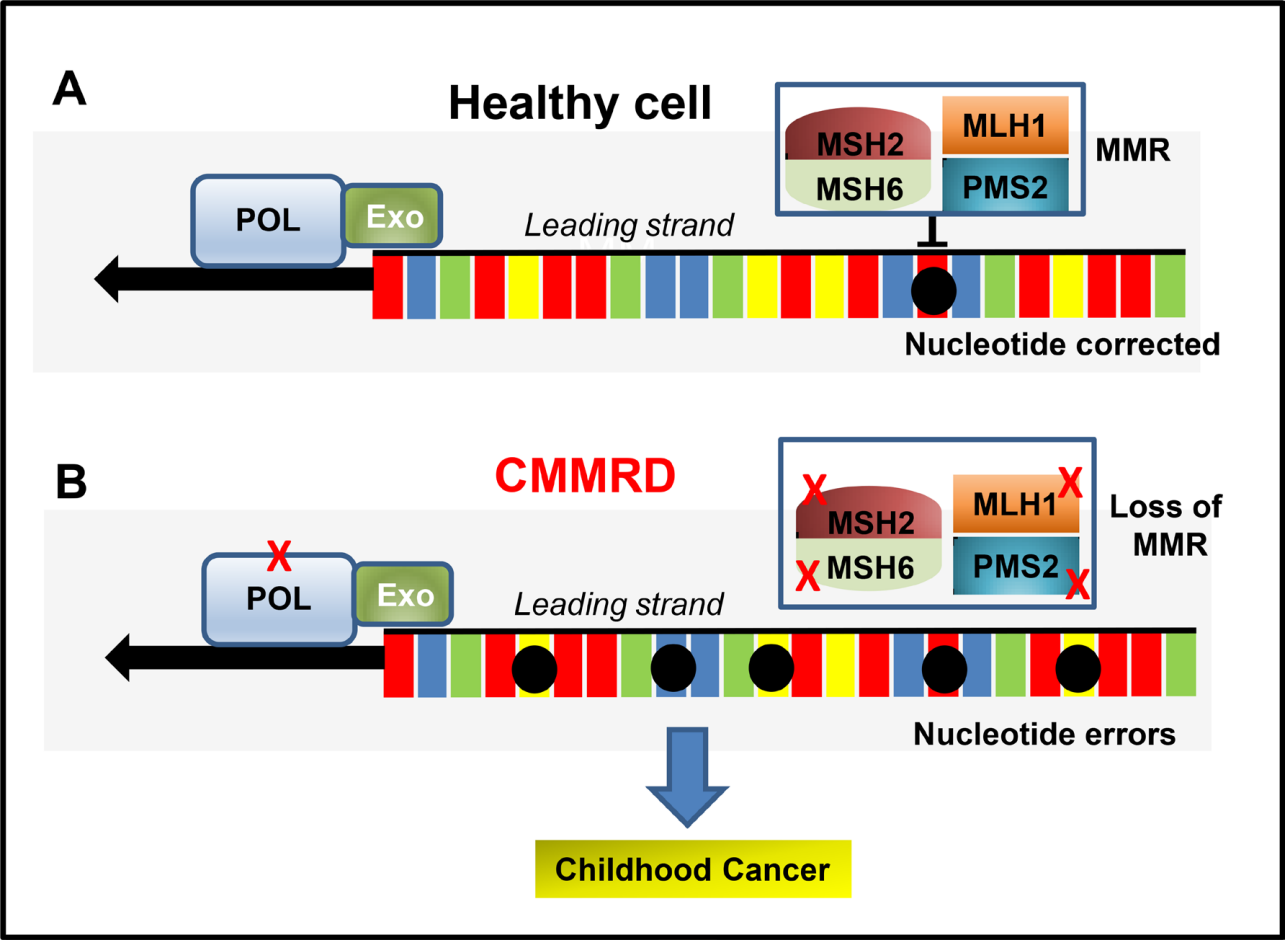
(**A**) Schematic of MMR in a healthy cell (adapted from [12]). The proofreading capability of the DNA polymerases (POL) and the MMR system recognises and prevent errors (black circles) during DNA replication. (**B**) CMMRD. Inherited MMR defects (X) that lead to a loss of MMR function/expression lead to accumulated mutations and a predisposition to cancer during adulthood. When a combination of mutations affect POL and MMR function, the accumulation of mutations become more rapid and the onset of cancer occurs in young children (CMMRD).

Constitutional Mismatch Repair Deficiency (CMMRD) (also known as Biallelic Mismatch Repair Deficiency: BMMRD) is a hereditary cancer predisposition that presents in infancy or young adulthood at an incidence of approximately 1 per million patients [10]. CMMRD occurs as a result of mutations in well characterised MMR genes including mutS homolog 2 (MSH2); mutL homolog 1 (MLH1); mutS homolog 6 (MSH6); post-meiotic segregation increased 2 (PMS2); and post-meiotic segregation increased 1 (PMS1) [11, 12]. The major function of these genes are to eliminate the mismatch of base-base insertions and deletions that occur as a consequence of DNA polymerase errors during DNA synthesis (Figure 1) [13]. Single-nucleotide variations (SNV) result from errors during base pair incorporation whilst slippages of the polymerase result in insertions and deletions [14]. MMR genes act to promote genome stabilization through correcting these errors, ensuring the fidelity of genetic recombination and the initiation of apoptosis in response to DNA damage (Figure 1). MMR genes have been extensively studied and their contribution to disease has been reviewed in several reports [11, 15].

**Figure 2:**
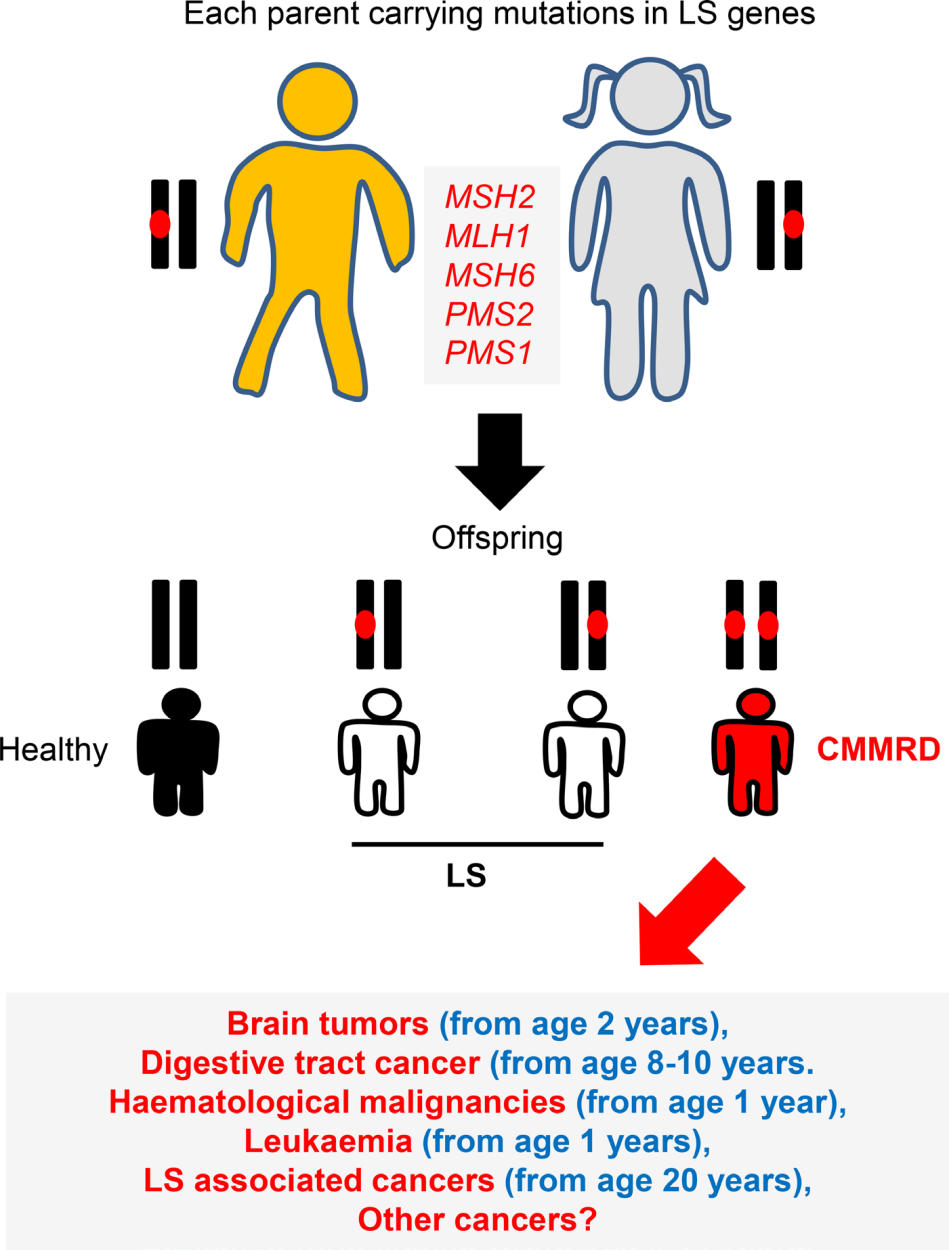
CMMRD genetics LS is an autosomal dominant disorder caused by defects in one of DNA MMR genes. Siblings of two parents with LS can develop CMMRD (biallelic MMR mutations). The spectrum of cancers observed for CMMRD are more severe than those found in LS. Up to 50% of children develop brain tumours, around 50% digestive tract cancers and approximately 33% develop haematological malignancies.

Heterozygous (monoallelic) mutations in MMR genes can impair MMR functionality resulting in a cancer condition termed Lynch Syndrome (LS), previously known as hereditary non-polyposis colorectal cancer (HNPCC) syndrome [9, 11, 16–19]. LS is characterized by gastrointestinal and genitourinary cancers during adulthood and represents 1–7% of all cases of colorectal cancer (CRC) [9, 11, 18]. For LS to be defined, germline mutations in at least one of the repair genes must be identified [17]. In contrast, biallelic germline mutations in the MMR genes that cause LS leads to CMMRD. The estimated carrier frequencies for mutations in the MMR-genes are 1 in 1946 for MLH1, 1 in 2841 for MSH2, 1 in 758 for MSH6 and 1 in 714 for PMS2 [20, 21]. If both parents have LS, the CMMRD risk to the siblings is 25% chance of having CMMRD, 25% chance of no LS mutations and a 50% chance of LS (Figure 2). Individuals with CMMRD develop a large variety of malignant neoplasms during early life with the majority of sufferer’s failing to reaching adulthood [8, 20, 22–27].

## HISTORY

Nearly twenty years ago, case reports presented a phenotype of offspring from consanguineous marriages within LS families (both carried MLH1 mutations) [28]. The offspring developed malignancies during early childhood. Of note, the individuals displayed clinical features reminiscent of neurofibromatosis type 1 (NF1) (tumor formation on nerve tissues), now commonly associated with CMMRD [28]. Since those studies, close to 200 paediatric and young adult CMMRD cancer cases have been reported in at least one of the MMR genes involved in LS [10, 29]. Contrary to traditional LS, CMMRD patients lack expression of the MMR protein(s) in both cancer and normal tissue (27). This recessively inherited condition is now fully recognised as a distinct childhood cancer predisposition syndrome [1, 30–33].

Although unproven at the molecular level, Jacques Turcot is attributed to have described the first cases of CMMRD in siblings with colorectal adenomatous polyps, colorectal carcinoma and malignant brain tumours [34]. ‘Turcot syndrome’ classically refers to a combination of colorectal polyposis and primary tumors of the Central Nervous System (CNS) [34–47]. CMMRD was referred to under Turcot syndrome for many years, until it was noted that this definition was too restrictive, as the manifestation of CMMRD also includes early-onset hematologic malignancies and cafe-au-lait spots suggestive of NF1 [31, 48]. Other non-neoplastic features have now emerged that are indicative of CMMRD in paediatric cancer patients, but until recently the CMMRD diagnostic criteria was lacking [49, 50]. The ability to accurately diagnose CMMRD is of critical importance to patient treatment and care, particularly in Arab and developing countries due to their high prevalence of consanguinity and increased susceptibility to this syndrome.

## CMMRD DIAGNOSIS

The rapid identification of CMMRD is crucial for patient management and for afflicted family members. However, given the complex nature of CMMRD, diagnosis is often delayed or in some instances, not-stated. A major reason for the lack of CMMRD awareness amongst pediatric oncologists can be explained by the diagnostic difficulties that result from the lack of clear disease-specific clinical features that combine the full spectrum of CMMRD tumors. This has, on occasion, resulted in the refusal of standard CMMRD treatment due to families being unconvinced by the initial diagnostic criteria [5]. To address this issue, a newly established European consortium named “Care for CMMRD” developed a scoring system of clinical criteria used to confirm CMMRD diagnosis (summarized in Table 1). However, these guidelines are however difficult to follow due to the rarity of CMMRD cases and the diversity of cancer presentation [11, 12]. Various clinical characteristics that suggest CMRRD include any child or young adult with an LS associated tumor, hypermutated tumors, adenomatous polyposis, pediatric cancer in the setting of consanguinity, loss of MMR protein expression in normal and tumor tissues, and café au lait spots without NF1 diagnosis. It is also recommended to consider CMMRD syndrome in individuals with brain cancer, leukemia or lymphoma that lack a history of radiation exposure. As with the majority of autosomal recessive diseases, the index case of CMMRD typically lacks a family history of cancer that would raise a suspicion of CMMRD. Many of the cases identified to date are thus ascertained only after another sibling becomes affected by cancer.

**Table 1:** Diagnostic scoring criteria for CMMRD from the European consortium “Care for CMMRD” [10]

Indications for CMMRD-Testing	More than 3 points
Maligancies or pre-malignancies: one is mandatory. If more than one is present add points	
LS carcinoma^*^ at age less than 25 years	3 points
Multiple bowel adenomas at age less than 25 years and absence of APC/MUTYH or a single grade dysplasic adenoma (also at age less than 25 years).	3 points
WHO grade III or IV glioma at age less than 25 years	2 points
NHL of T-cell lineage or sPNET at age less than 18 years	2 points
Any malignancy in a patient under 18 years	1 point

Additional features: if more than one of the following are present add points	
Clinical NF1 diagnosis or more than 2 hyper/hypo-skin pigmentations (greater than 1 cm)	2 points
Diagnosis of LS in a 1st and/or 2nd degree relative	2 points
LS carcinoma^*^, high grade glioma, sPNET, or NHL	1 point
Sibling with a childhood cancer	1 point
Multiple pilomatricomas present	2 points
One pilomatricoma present	1 point
Agenesis of the corpus callosum or non-therapy induced cavernoma	1 point
Consanguineous parents^#^	1 point
Deficiency/reduced levels of IgG2/4 and or IgA	1 point

LS cancers: CRC, endometrial, small bowel, renal pelvis, ureter, biliary tract, bladder, stomach.

^#^more common in Arab/Developing countries.

The evolution of sequencing technologies has benefited diagnosis as the rapid detection of characteristic CMMRD mutational patterns can be obtained from patient blood and tumor samples [51, 52]. This can be used simultaneously to facilitate downstream germline testing [25, 51, 52] and be used to offer genetic counseling to families with “at-risk” siblings. [8]. This information can also provide personalized targeted treatment programs, once the genetic basis of the CMMRD tumor is understood [52, 53].

## CMMRD TUMOR SPECTRUM

The prognosis of CMMRD is poor; current statistics suggest over 50% of patients develop malignant brain tumours, 40% develop digestive tract tumours and 30% develop haematological malignancies, all during childhood [27]. The most frequent CMMRD cancers are brain gliomas (diagnosed at an average age of 9.5 years), non-Hodgkin’s lymphomas (diagnosed at 5 years) and colorectal cancers (CRCs) (diagnosed at 16 years) [10]. The cancer spectrum is related to the nature of the MMR gene mutated; patients with MSH6 and/or PMS2 mutations develop brain tumours within 10 years of life and over 40% of patients homozygous for PMS2 mutations develop second primary malignancies [29]. By comparison, patients homozygous for MLH1/MSH2 mutations are less likely to develop second primary malignancies (22%). This difference is because patients with homozygous PMS2 mutations often survive their first malignancy, unlike MLH1/MSH2 patients who develop more aggressive haematological malignancies [10]. Consanguinity of the parents and/or homozygosity for a founder mutation is observed in over 50% of CMMRD-cancers [10, 17] the most common of which are reviewed in [21].

## CMMRD SURVEILLANCE

CMMRD tumors in the CNS are often observed during infancy, and as such MRI scanning is generally performed in the first two years of life. Brain MRI is recommended at diagnosis and every 6 months thereafter [54, 55]. Repeated CT scanning of the brain is not recommended due to the possible induction of tumours due to radiation [54]. For digestive tract cancers (including CRC), colonoscopy is used for surveillance. Colonic polyps have been reported in CMMRD patients as early as 6 years of age, so surveillance is generally initiated at this age [56]. Once polyps are identified, colonoscopy is performed every 6 months under general anaesthesia. Flat and non-polypoid lesions can be missed so colonoscopy is recommended for their detection [27, 54]. Colectomy is considered in patients with high-grade dysplasia as these present a significant risk of carcinoma [54]. Small bowel polyps develop later in life so upper endoscopy is recommended at 8 years of age in these patients. For the detection of duodenal cancers, upper GI endoscopy is performed at the same time as colonoscopy. Lymphoid and other hematologic malignancies represent the third most common malignancies in CMMRD, but effective tools for diagnosis are still lacking [57]. Information on the natural history of CMMRD lymphomas is sparse and the natural course of the disease may differ from sporadic cases [54]. These typically present with tumours and clinical manifestations a month prior to diagnosis. Standard surveillance is performed every six months including repeated blood counts and abdominal ultrasounds [27, 54]. This strategy is useful to assess the natural history of the lymphoma.

A modification to current protocols now includes the implementation of whole body MRI to be implemented once a year at 6 years of age (when anaesthesia is not needed). This is not recommended as a replacement for ultrasound and brain MRI, but may present benefits for CMMRD diagnosis as the spectrum of CMMRD cancers continues to increase [54].

## CURRENT CMMRD TREATMENT

Children with CMMRD have a high risk of developing multiple cancers and early diagnosis does not guarantee detection at a curable stage [49, 50]. Preventive treatment strategies would represent a major advance for CMMRD therapy and the benefits of novel long-term conventional therapies such as acetylsalicylic acid (aspirin) are emerging [58]. As CMMRD is rare, information on its optimal therapeutic strategies are limited and current knowledge of treatment regimens and their outcomes are limited to individual case reports, often with variable disease phenotypes [29, 36, 39, 42, 46, 59–70]. Chemotherapy remains a frontline treatment, but toxicity is a major issue in children. Selection of appropriate chemotherapy drugs [71–74] is generally based on their toxicity profile [75, 76] and the knowledge of tumor resistance [71].

## CHEMOTHERAPY

Many frequently used chemotherapeutic agents require a functional MMR system to initiate tumor damage. Accordingly, MMR deficient cells are frequently resistant to chemotherapeutics [77–95]. This includes resistance to mercaptopurine and temozolomide, drugs commonly used to treat hematopoietic and glioma cancers, respectively [55, 96–115]. MMR resistance is exemplified by temozolomide, the drug of choice for glioblastomas multiforme (GBM), a highly malignant CMMRD-brain tumor [24, 54, 116, 117]. The response to temozolomide is limited in CMMRD-related GBM and its use is now avoided due to its known ability to increase the accumulation of somatic mutations in patients, increasing the risk of secondary tumors [15, 71, 118]. No obvious lack of efficacy of other therapeutic agents such as alkylating agents has been reported, but effective chemotherapeutic therapies for CMMRD cancer are still lacking, and new effective therapies are urgently required [54].

## CMMRD IMMUNOTHERAPY

The ultrahypermutation phenotype (≥ 100 mut/Mb compared to <10/MB in other childhood cancers) of CMMRD tumors does offer some opportunities for new approaches to treatment [117, 119]. When microsatellites in gene-encoding regions are mutated in CMMRD, the numerous frameshifts lead to the production of truncated and functionally inactive proteins that are frequently processed into mutanome-derived epitopes (termed neoantigens) that are presented to cytotoxic T lymphocytes (CTLs) [71]. Mutations in the exonuclease domain of the catalytic subunit of DNA polymerase epsilon (POLE) also exhibit such ultra mutated genomes (Figure.1). Neoantigen loads in CMMRD are substantially higher than in other cancer patients without the condition making these tumors more likely to be recognized by the immune system [120, 121]. Tumors that are recognized by the immune system have an improved prognosis. For example, greater densities of tumor-infiltrating lymphocytes are observed in cases of CRC with MSI [56, 122]. This improves their prognosis when compared to microsatellite stable CRCs and alters the response to chemotherapeutics. The use of NGS technologies can therefore guide treatment regimens and identify hypermutated tumors (≥100 mut/Mb) most likely to respond to immunotherapy. Meier and coworkers also recently performed signature extractions from 215 human CRC cases and 289 gastric adenocarcinomas revealing three novel MMR-associated signatures that strongly discriminate MS stable and unstable tumors, the knowledge of which can dictate treatment planning following identification [123].

## CHECKPOINT INHIBITORS

In the presence of malignant tumors, immunoreactivity becomes compromised due to tumor induced immunosuppression. PD-L1 is overexpressed in many cancers and acts as a binding site for PD1. Binding of PD1 to PD-L1 within the tumor activates PD1 signalling, which in turn inhibits T cell activation allowing the tumor to evade immune attack [124, 125]. Inhibiting the interaction of PD1 and PD-L1 can thus enhance the anti-cancer T cell response and promote anti-tumor activity. This knowledge has been used to develop immunotherapies termed checkpoint inhibitors, that counteract the actions of proteins that impede the immune response to cancer. Blocking PD-1 in CMMRD tumors produces a significant clinical response and CMMRD tumors are more responsive to PD-1 blockers than MMR proficient tumors [117, 124, 125]. When PD1- blockers were used to treat children with CMMRD with recurrent GBM, shrinking of tumors through MRI was observed, indicating a successful clinical response [117]. Other recent data have demonstrated the effectiveness of checkpoint inhibitors in the treatment of some non-Hodgkin lymphomas [57]. This is of interest to CMMRD patients that often have non-Hodgkin’s lymphoma in addition to other cancers [26].

## NEOANTIGEN VACCINATION

Vaccination with neoantigens is another promising approach for CMMRD cancer and neoantigen-loaded cell vaccinations are in clinical trials for CRC patients with MSI. Preliminary data from these trials suggests they are safe and well tolerated [126, 127]. Strong immune responses against neoantigen vaccines have been observed in LS patients that already show neoantigen-specific immune responses [126]. This holds promise for the use of adjuvant or preventive neoantigen-based vaccinations for CMMRD. However, the limited number of CMMRD patients means the identification of commonly mutated microsatellites is challenging. In addition, CMMRD vaccinations must be approached with caution since all cells of a CMMRD patient are MMR deficient [17–19] leading to a risk of autoimmune disease. It is likely that the combination of check-point inhibitors and neoantigen vaccination will hold the most promise for CMMRD. Indeed, our own recent studies highlight the benefits of incorporating genomic and/or molecular testing for CMMRD into routine paediatric oncology, whereby clinical care can identify a subset of patients likely to benefit from targeted treatment regimens, dependent on their MMR mutation status [53].

## SUMMARY

CMMRD has emerged a rare childhood cancer syndrome and as such, consistency regarding its diagnosis and optimal treatment strategies have been challenging. The spectrum of CMMRD-associated tumours is broad and as sequencing technologies and thus diagnosis evolves, these are likely to expand further. The severity of CCMRD is well understood; CMMRD-patients possess a high risk of multiple cancers during childhood and typically do not survive to later life. Urgent CMMRD therapies are therefore required and hope in this area has been provided through immunotherapy interventions, including check-point-inhibitors and/or neoantigen vaccinations. The promise of anti-cancer immunotherapy must now be combined with our knowledge of the underlying genetic basis of CMMRD-tumors to produce personalised and targeted CMMRD treatment regimens. These will benefit current CMMRD patients, and future CMMRD-afflicted children.

## Abbreviations

MMR: Mismatch repair
CMMRD: Constitutional Mismatch Repair Deficiency
LS: Lynch Syndrome
HNPCC: Hereditary Non-Polyposis Colorectal Cancer
CRC: Colorectal Cancer
BMMRD: Biallelic Mismatch Repair Deficiency
MSH2: mutS homolog 2
MLH1: mutL homolog 1
MSH6: mutS homolog 6
PMS2: post-meiotic segregation increased 2
PMS1: post-meiotic segregation increased 1
SNV: single-nucleotide variations
MSI: Microsatellite Instability
NF1: Neurofibromatosis type 1
GMB: glioblastoma multiforme
CTLs: Cytotoxic T lymphocytes
PD1: Programmed Cell Death Protein 1
